# Supplementary material for: Analysis of eligibility criteria clusters based on large language models for clinical trial design
Source: J Am Med Inform Assoc. 2024 Dec 26;32(3):447–58. doi: 10.1093/jamia/ocae311 (PMC11833473; doi:10.1093/jamia/ocae311)
Supplement: ocae311_Supplementary_Data [file ocae311_supplementary_data.zip › ocae311_Supplementary_Data/Supplementary Information S1.pdf]

S1 – Dataset – Descriptive Statistics

Total	**Total EC Count**	**2071643**	**Total CT Count**	**98376**
	Phase 1 EC Count	734574 (35.46%)	Phase 1 CT Count	30223 (30.72%)
	Phase 2 EC Count	878948 (42.43%)	Phase 2 CT Count	36863 (37.47%)
	Phase 3 EC Count	403262 (19.47%)	Phase 3 CT Count	22653 (23.03%)
	Phase 4 EC Count	245423 (11.85%)	Phase 4 CT Count	16778 (17.05%)
	ECs per CT	21.06 ± 15.59		
C01	**Total EC Count**	**195738 (9.45%)**	**Total CT Count**	**9434 (9.59%)**
	Phase 1 EC Count	54995 (28.10%)	Phase 1 CT Count	2044 (21.67%)
	Phase 2 EC Count	75422 (38.53%)	Phase 2 CT Count	3419 (36.24%)
	Phase 3 EC Count	51759 (26.44%)	Phase 3 CT Count	2799 (29.67%)
	Phase 4 EC Count	31029 (15.85%)	Phase 4 CT Count	1963 (20.81%)
	ECs per CT	20.75 ± 15.60		
C04	**Total EC Count**	**740945 (35.77%)**	**Total CT Count**	**24998 (25.41%)**
	Phase 1 EC Count	290313 (39.18%)	Phase 1 CT Count	9136 (36.55%)
	Phase 2 EC Count	456154 (61.56%)	Phase 2 CT Count	14815 (59.26%)
	Phase 3 EC Count	80967 (10.93%)	Phase 3 CT Count	3387 (13.55%)
	Phase 4 EC Count	12728 (1.72%)	Phase 4 CT Count	816 (3.26%)
	ECs per CT	29.64 ± 18.18		
C05	**Total EC Count**	**76898 (3.71%)**	**Total CT Count**	**4232 (4.30%)**
	Phase 1 EC Count	14561 (18.94%)	Phase 1 CT Count	677 (16.00%)
	Phase 2 EC Count	29785 (38.73%)	Phase 2 CT Count	1462 (34.55%)
	Phase 3 EC Count	24983 (32.49%)	Phase 3 CT Count	1464 (34.59%)
	Phase 4 EC Count	13793 (17.94%)	Phase 4 CT Count	942 (22.26%)
	ECs per CT	18.17 ± 14.34		
C06	**Total EC Count**	**222708 (10.75%)**	**Total CT Count**	**9390 (9.55%)**
	Phase 1 EC Count	70204 (31.52%)	Phase 1 CT Count	2583 (27.51%)
	Phase 2 EC Count	118326 (53.13%)	Phase 2 CT Count	4546 (48.41%)
	Phase 3 EC Count	39965 (17.95%)	Phase 3 CT Count	2051 (21.84%)
	Phase 4 EC Count	17715 (7.95%)	Phase 4 CT Count	1130 (12.03%)
	ECs per CT	23.72 ± 16.89		
C07	**Total EC Count**	**23422 (1.13%)**	**Total CT Count**	**1301 (1.32%)**
	Phase 1 EC Count	4294 (18.33%)	Phase 1 CT Count	189 (14.53%)
	Phase 2 EC Count	13395 (57.19%)	Phase 2 CT Count	663 (50.96%)
	Phase 3 EC Count	5500 (23.48%)	Phase 3 CT Count	378 (29.05%)
	Phase 4 EC Count	2932 (12.52%)	Phase 4 CT Count	249 (19.14%)

	ECs per CT	18.00 ± 14.24		
C08	**Total EC Count**	**221358 (10.69%)**	**Total CT Count**	**9454 (9.61%)**
	Phase 1 EC Count	58335 (26.35%)	Phase 1 CT Count	2069 (21.88%)
	Phase 2 EC Count	108523 (49.03%)	Phase 2 CT Count	4310 (45.59%)
	Phase 3 EC Count	53320 (24.09%)	Phase 3 CT Count	2601 (27.51%)
	Phase 4 EC Count	22037 (9.96%)	Phase 4 CT Count	1332 (14.09%)
	ECs per CT	23.41 ± 16.75		
C09	**Total EC Count**	**26099 (1.26%)**	**Total CT Count**	**1249 (1.27%)**
	Phase 1 EC Count	4651 (17.82%)	Phase 1 CT Count	181 (14.49%)
	Phase 2 EC Count	10835 (41.52%)	Phase 2 CT Count	478 (38.27%)
	Phase 3 EC Count	8337 (31.94%)	Phase 3 CT Count	437 (34.99%)
	Phase 4 EC Count	4480 (17.17%)	Phase 4 CT Count	254 (20.34%)
	ECs per CT	20.90 ± 15.82		
C10	**Total EC Count**	**192647 (9.30%)**	**Total CT Count**	**9706 (9.87%)**
	Phase 1 EC Count	49811 (25.86%)	Phase 1 CT Count	2077 (21.40%)
	Phase 2 EC Count	86012 (44.65%)	Phase 2 CT Count	4013 (41.35%)
	Phase 3 EC Count	50585 (26.26%)	Phase 3 CT Count	2843 (29.29%)
	Phase 4 EC Count	27090 (14.06%)	Phase 4 CT Count	1730 (17.82%)
	ECs per CT	19.85 ± 14.51		
C11	**Total EC Count**	**48933 (2.36%)**	**Total CT Count**	**2914 (2.96%)**
	Phase 1 EC Count	11450 (23.40%)	Phase 1 CT Count	536 (18.39%)
	Phase 2 EC Count	20773 (42.45%)	Phase 2 CT Count	1176 (40.36%)
	Phase 3 EC Count	13826 (28.25%)	Phase 3 CT Count	882 (30.27%)
	Phase 4 EC Count	9116 (18.63%)	Phase 4 CT Count	657 (22.55%)
	ECs per CT	16.79 ± 13.26		
C12	**Total EC Count**	**223205 (10.77%)**	**Total CT Count**	**9825 (9.99%)**
	Phase 1 EC Count	71061 (31.84%)	Phase 1 CT Count	2471 (25.15%)
	Phase 2 EC Count	107746 (48.27%)	Phase 2 CT Count	4203 (42.78%)
	Phase 3 EC Count	41661 (18.66%)	Phase 3 CT Count	2290 (23.31%)
	Phase 4 EC Count	24899 (11.16%)	Phase 4 CT Count	1757 (17.88%)
	ECs per CT	22.72 ± 16.84		
C14	**Total EC Count**	**180401 (8.71%)**	**Total CT Count**	**9027 (9.18%)**
	Phase 1 EC Count	42693 (23.67%)	Phase 1 CT Count	1674 (18.54%)
	Phase 2 EC Count	73691 (40.85%)	Phase 2 CT Count	3266 (36.18%)
	Phase 3 EC Count	45686 (25.32%)	Phase 3 CT Count	2574 (28.51%)
	Phase 4 EC Count	36050 (19.98%)	Phase 4 CT Count	2280 (25.26%)
	ECs per CT	19.98 ± 14.31		
C15	**Total EC Count**	**200329 (9.67%)**	**Total CT Count**	**7496 (7.62%)**

	Phase 1 EC Count	81186 (40.53%)	Phase 1 CT Count	2626 (35.03%)
	Phase 2 EC Count	116503 (58.16%)	Phase 2 CT Count	4190 (55.90%)
	Phase 3 EC Count	25141 (12.55%)	Phase 3 CT Count	1241 (16.56%)
	Phase 4 EC Count	6423 (3.21%)	Phase 4 CT Count	441 (5.88%)
	ECs per CT	26.72 ± 17.09		
C16	**Total EC Count**	**75839 (3.66%)**	**Total CT Count**	**4167 (4.24%)**
	Phase 1 EC Count	21179 (27.93%)	Phase 1 CT Count	1026 (24.62%)
	Phase 2 EC Count	39208 (51.70%)	Phase 2 CT Count	2007 (48.16%)
	Phase 3 EC Count	18398 (24.26%)	Phase 3 CT Count	1173 (28.15%)
	Phase 4 EC Count	6575 (8.67%)	Phase 4 CT Count	486 (11.66%)
	ECs per CT	18.20 ± 13.28		
C17	**Total EC Count**	**194765 (9.40%)**	**Total CT Count**	**8471 (8.61%)**
	Phase 1 EC Count	51157 (26.27%)	Phase 1 CT Count	1870 (22.08%)
	Phase 2 EC Count	101503 (52.12%)	Phase 2 CT Count	4052 (47.83%)
	Phase 3 EC Count	43765 (22.47%)	Phase 3 CT Count	2243 (26.48%)
	Phase 4 EC Count	16326 (8.38%)	Phase 4 CT Count	999 (11.79%)
	ECs per CT	22.99 ± 17.35		
C18	**Total EC Count**	**148559 (7.17%)**	**Total CT Count**	**8428 (8.57%)**
	Phase 1 EC Count	39231 (26.41%)	Phase 1 CT Count	2000 (23.73%)
	Phase 2 EC Count	46602 (31.37%)	Phase 2 CT Count	2400 (28.48%)
	Phase 3 EC Count	43323 (29.16%)	Phase 3 CT Count	2657 (31.53%)
	Phase 4 EC Count	29753 (20.03%)	Phase 4 CT Count	1911 (22.67%)
	ECs per CT	17.63 ± 12.68		
C19	**Total EC Count**	**147259 (7.11%)**	**Total CT Count**	**7062 (7.18%)**
	Phase 1 EC Count	42538 (28.89%)	Phase 1 CT Count	1727 (24.45%)
	Phase 2 EC Count	62238 (42.26%)	Phase 2 CT Count	2535 (35.90%)
	Phase 3 EC Count	34038 (23.11%)	Phase 3 CT Count	1952 (27.64%)
	Phase 4 EC Count	21654 (14.70%)	Phase 4 CT Count	1373 (19.44%)
	ECs per CT	20.85 ± 15.60		
C20	**Total EC Count**	**285730 (13.79%)**	**Total CT Count**	**11906 (12.10%)**
	Phase 1 EC Count	99853 (34.95%)	Phase 1 CT Count	3430 (28.81%)
	Phase 2 EC Count	141240 (49.43%)	Phase 2 CT Count	5493 (46.14%)
	Phase 3 EC Count	53629 (18.77%)	Phase 3 CT Count	2765 (23.22%)
	Phase 4 EC Count	24627 (8.62%)	Phase 4 CT Count	1451 (12.19%)
	ECs per CT	24.00 ± 16.81		
C21	**Total EC Count**	**10 (0.00%)**	**Total CT Count**	**1 (0.00%)**
	Phase 1 EC Count	0 (0.00%)	Phase 1 CT Count	0 (0.00%)
	Phase 2 EC Count	10 (100.00%)	Phase 2 CT Count	1 (100.00%)

	Phase 3 EC Count	0 (0.00%)	Phase 3 CT Count	0 (0.00%)
	Phase 4 EC Count	0 (0.00%)	Phase 4 CT Count	0 (0.00%)
	ECs per CT	10.00 ± 0.00		
C22	**Total EC Count**	**1688 (0.08%)**	**Total CT Count**	**39 (0.04%)**
	Phase 1 EC Count	973 (57.64%)	Phase 1 CT Count	21 (53.85%)
	Phase 2 EC Count	745 (44.14%)	Phase 2 CT Count	18 (46.15%)
	Phase 3 EC Count	42 (2.49%)	Phase 3 CT Count	2 (5.13%)
	Phase 4 EC Count	8 (0.47%)	Phase 4 CT Count	1 (2.56%)
	ECs per CT	43.28 ± 23.46		
C23	**Total EC Count**	**480769 (23.21%)**	**Total CT Count**	**24244 (24.64%)**
	Phase 1 EC Count	113570 (23.62%)	Phase 1 CT Count	4534 (18.70%)
	Phase 2 EC Count	212171 (44.13%)	Phase 2 CT Count	9563 (39.44%)
	Phase 3 EC Count	118839 (24.72%)	Phase 3 CT Count	6785 (27.99%)
	Phase 4 EC Count	80829 (16.81%)	Phase 4 CT Count	5496 (22.67%)
	ECs per CT	19.83 ± 14.97		
C24	**Total EC Count**	**716 (0.03%)**	**Total CT Count**	**30 (0.03%)**
	Phase 1 EC Count	182 (25.42%)	Phase 1 CT Count	6 (20.00%)
	Phase 2 EC Count	318 (44.41%)	Phase 2 CT Count	12 (40.00%)
	Phase 3 EC Count	243 (33.94%)	Phase 3 CT Count	12 (40.00%)
	Phase 4 EC Count	73 (10.20%)	Phase 4 CT Count	3 (10.00%)
	ECs per CT	23.87 ± 13.03		
C25	**Total EC Count**	**31114 (1.50%)**	**Total CT Count**	**1641 (1.67%)**
	Phase 1 EC Count	8855 (28.46%)	Phase 1 CT Count	408 (24.86%)
	Phase 2 EC Count	14873 (47.80%)	Phase 2 CT Count	772 (47.04%)
	Phase 3 EC Count	5917 (19.02%)	Phase 3 CT Count	338 (20.60%)
	Phase 4 EC Count	4719 (15.17%)	Phase 4 CT Count	305 (18.59%)
	ECs per CT	18.96 ± 12.68		
C26	**Total EC Count**	**21110 (1.02%)**	**Total CT Count**	**1290 (1.31%)**
	Phase 1 EC Count	4334 (20.53%)	Phase 1 CT Count	206 (15.97%)
	Phase 2 EC Count	9359 (44.33%)	Phase 2 CT Count	508 (39.38%)
	Phase 3 EC Count	5396 (25.56%)	Phase 3 CT Count	352 (27.29%)
	Phase 4 EC Count	4709 (22.31%)	Phase 4 CT Count	387 (30.00%)
	ECs per CT	16.36 ± 11.70		
D01	**Total EC Count**	**63773 (3.08%)**	**Total CT Count**	**2745 (2.79%)**
	Phase 1 EC Count	20089 (31.50%)	Phase 1 CT Count	697 (25.39%)
	Phase 2 EC Count	32964 (51.69%)	Phase 2 CT Count	1277 (46.52%)
	Phase 3 EC Count	12303 (19.29%)	Phase 3 CT Count	634 (23.10%)
	Phase 4 EC Count	5660 (8.88%)	Phase 4 CT Count	426 (15.52%)

	ECs per CT	23.23 ± 15.43		
D02	**Total EC Count**	**753350 (36.36%)**	**Total CT Count**	**35303 (35.89%)**
	Phase 1 EC Count	229359 (30.45%)	Phase 1 CT Count	8771 (24.84%)
	Phase 2 EC Count	329738 (43.77%)	Phase 2 CT Count	13019 (36.88%)
	Phase 3 EC Count	155189 (20.60%)	Phase 3 CT Count	8471 (24.00%)
	Phase 4 EC Count	112984 (15.00%)	Phase 4 CT Count	8020 (22.72%)
	ECs per CT	21.34 ± 15.65		
D03	**Total EC Count**	**694545 (33.53%)**	**Total CT Count**	**32285 (32.82%)**
	Phase 1 EC Count	222788 (32.08%)	Phase 1 CT Count	8748 (27.10%)
	Phase 2 EC Count	292863 (42.17%)	Phase 2 CT Count	11459 (35.49%)
	Phase 3 EC Count	142259 (20.48%)	Phase 3 CT Count	7792 (24.14%)
	Phase 4 EC Count	98694 (14.21%)	Phase 4 CT Count	6804 (21.07%)
	ECs per CT	21.51 ± 15.66		
D04	**Total EC Count**	**257170 (12.41%)**	**Total CT Count**	**12347 (12.55%)**
	Phase 1 EC Count	72250 (28.09%)	Phase 1 CT Count	2876 (23.29%)
	Phase 2 EC Count	107162 (41.67%)	Phase 2 CT Count	4465 (36.16%)
	Phase 3 EC Count	61378 (23.87%)	Phase 3 CT Count	3269 (26.48%)
	Phase 4 EC Count	40605 (15.79%)	Phase 4 CT Count	2781 (22.52%)
	ECs per CT	20.83 ± 15.36		
D05	**Total EC Count**	**3972 (0.19%)**	**Total CT Count**	**248 (0.25%)**
	Phase 1 EC Count	485 (12.21%)	Phase 1 CT Count	25 (10.08%)
	Phase 2 EC Count	1159 (29.18%)	Phase 2 CT Count	67 (27.02%)
	Phase 3 EC Count	1531 (38.54%)	Phase 3 CT Count	89 (35.89%)
	Phase 4 EC Count	1063 (26.76%)	Phase 4 CT Count	85 (34.27%)
	ECs per CT	16.02 ± 10.77		
D06	**Total EC Count**	**76750 (3.70%)**	**Total CT Count**	**4222 (4.29%)**
	Phase 1 EC Count	18804 (24.50%)	Phase 1 CT Count	981 (23.24%)
	Phase 2 EC Count	26937 (35.10%)	Phase 2 CT Count	1271 (30.10%)
	Phase 3 EC Count	21345 (27.81%)	Phase 3 CT Count	1219 (28.87%)
	Phase 4 EC Count	15290 (19.92%)	Phase 4 CT Count	1045 (24.75%)
	ECs per CT	18.18 ± 13.62		
D08	**Total EC Count**	**33703 (1.63%)**	**Total CT Count**	**1476 (1.50%)**
	Phase 1 EC Count	8078 (23.97%)	Phase 1 CT Count	291 (19.72%)
	Phase 2 EC Count	17556 (52.09%)	Phase 2 CT Count	714 (48.37%)
	Phase 3 EC Count	9105 (27.02%)	Phase 3 CT Count	433 (29.34%)
	Phase 4 EC Count	2749 (8.16%)	Phase 4 CT Count	192 (13.01%)
	ECs per CT	22.83 ± 15.46		
D09	**Total EC Count**	**86199 (4.16%)**	**Total CT Count**	**3741 (3.80%)**

	Phase 1 EC Count	25787 (29.92%)	Phase 1 CT Count	975 (26.06%)
	Phase 2 EC Count	41835 (48.53%)	Phase 2 CT Count	1590 (42.50%)
	Phase 3 EC Count	19895 (23.08%)	Phase 3 CT Count	967 (25.85%)
	Phase 4 EC Count	8635 (10.02%)	Phase 4 CT Count	603 (16.12%)
	ECs per CT	23.04 ± 16.30		
D10	**Total EC Count**	**53361 (2.58%)**	**Total CT Count**	**3132 (3.18%)**
	Phase 1 EC Count	11977 (22.45%)	Phase 1 CT Count	547 (17.46%)
	Phase 2 EC Count	19733 (36.98%)	Phase 2 CT Count	962 (30.72%)
	Phase 3 EC Count	13026 (24.41%)	Phase 3 CT Count	879 (28.07%)
	Phase 4 EC Count	12560 (23.54%)	Phase 4 CT Count	954 (30.46%)
	ECs per CT	17.04 ± 13.05		
D12	**Total EC Count**	**342384 (16.53%)**	**Total CT Count**	**14800 (15.04%)**
	Phase 1 EC Count	93143 (27.20%)	Phase 1 CT Count	3474 (23.47%)
	Phase 2 EC Count	179013 (52.28%)	Phase 2 CT Count	6691 (45.21%)
	Phase 3 EC Count	71592 (20.91%)	Phase 3 CT Count	3691 (24.94%)
	Phase 4 EC Count	36654 (10.71%)	Phase 4 CT Count	2404 (16.24%)
	ECs per CT	23.13 ± 16.86		
D13	**Total EC Count**	**92524 (4.47%)**	**Total CT Count**	**3627 (3.69%)**
	Phase 1 EC Count	28312 (30.60%)	Phase 1 CT Count	929 (25.61%)
	Phase 2 EC Count	47792 (51.65%)	Phase 2 CT Count	1740 (47.97%)
	Phase 3 EC Count	17115 (18.50%)	Phase 3 CT Count	804 (22.17%)
	Phase 4 EC Count	10253 (11.08%)	Phase 4 CT Count	541 (14.92%)
	ECs per CT	25.51 ± 16.59		
D20	**Total EC Count**	**19868 (0.96%)**	**Total CT Count**	**780 (0.79%)**
	Phase 1 EC Count	6024 (30.32%)	Phase 1 CT Count	195 (25.00%)
	Phase 2 EC Count	10614 (53.42%)	Phase 2 CT Count	356 (45.64%)
	Phase 3 EC Count	3172 (15.97%)	Phase 3 CT Count	167 (21.41%)
	Phase 4 EC Count	2626 (13.22%)	Phase 4 CT Count	156 (20.00%)
	ECs per CT	25.47 ± 17.77		
D23	**Total EC Count**	**76585 (3.70%)**	**Total CT Count**	**3703 (3.76%)**
	Phase 1 EC Count	17794 (23.23%)	Phase 1 CT Count	681 (18.39%)
	Phase 2 EC Count	34561 (45.13%)	Phase 2 CT Count	1497 (40.43%)
	Phase 3 EC Count	19894 (25.98%)	Phase 3 CT Count	1071 (28.92%)
	Phase 4 EC Count	12090 (15.79%)	Phase 4 CT Count	800 (21.60%)
	ECs per CT	20.68 ± 15.65		
D25	**Total EC Count**	**3373 (0.16%)**	**Total CT Count**	**220 (0.22%)**
	Phase 1 EC Count	339 (10.05%)	Phase 1 CT Count	22 (10.00%)
	Phase 2 EC Count	846 (25.08%)	Phase 2 CT Count	55 (25.00%)

	Phase 3 EC Count	1307 (38.75%)	Phase 3 CT Count	75 (34.09%)
	Phase 4 EC Count	1010 (29.94%)	Phase 4 CT Count	78 (35.45%)
	ECs per CT	15.33 ± 10.01		
D26	**Total EC Count**	**33672 (1.63%)**	**Total CT Count**	**1886 (1.92%)**
	Phase 1 EC Count	7845 (23.30%)	Phase 1 CT Count	347 (18.40%)
	Phase 2 EC Count	8070 (23.97%)	Phase 2 CT Count	441 (23.38%)
	Phase 3 EC Count	10831 (32.17%)	Phase 3 CT Count	623 (33.03%)
	Phase 4 EC Count	8847 (26.27%)	Phase 4 CT Count	579 (30.70%)
	ECs per CT	17.85 ± 13.52		
D27	**Total EC Count**	**79844 (3.85%)**	**Total CT Count**	**3711 (3.77%)**
	Phase 1 EC Count	23521 (29.46%)	Phase 1 CT Count	820 (22.10%)
	Phase 2 EC Count	34641 (43.39%)	Phase 2 CT Count	1334 (35.95%)
	Phase 3 EC Count	17510 (21.93%)	Phase 3 CT Count	975 (26.27%)
	Phase 4 EC Count	12535 (15.70%)	Phase 4 CT Count	917 (24.71%)
	ECs per CT	21.52 ± 17.61		

Table A. Descriptive statistics of the dataset extracted from ClinicalTrials.gov. EC – Eligibility Criterion. Statistics are stratified by MeSH condition ID types (C01 – C26) and MeSH intervention ID types (D01 – D27). Numbers add up to more than 100% because most CT protocols have several condition ID types, intervention ID types, or even several phases. Moreover, the total EC or CT counts reported in this table are smaller than the numbers reported in Figure 3, A, B, and C, since the ECs used to create plots in these figures went through an additional filtering step: selected ECs came from CTs with at least one MeSH condition ID and one MeSH intervention ID with enough depth to produce the labels for experiment 1 (i.e., 3 levels for condition, 4 levels for intervention).
