# Supplementary material for: Analysis of eligibility criteria clusters based on large language models for clinical trial design
Source: J Am Med Inform Assoc. 2024 Dec 26;32(3):447–58. doi: 10.1093/jamia/ocae311 (PMC11833473; doi:10.1093/jamia/ocae311)
Supplement: ocae311_Supplementary_Data [file ocae311_supplementary_data.zip › ocae311_Supplementary_Data/Supplementary Information S2.pdf]

S2 – Optuna Hyper-Parameters

HDBSCAN Hyper-Parameters Fine-Tuned with Optuna

The hyper-parameters of HDBSCAN that were optimized with Optuna when clustering reduced eligibility criterion embeddings, and the range of possible values, were the following:

- For the first, “primary” cluster algorithm:
    - “max_cluster_size”: [0.01 * N, 0.1 * N]
    - “min_cluster_size”: [0.0 * N, 0.001 * N]
    - “min_samples”: min(128, [0.0 * N, 0.001 * N])
    - “alpha”: [0.1, 5.0]
    - “cluster_selection_method”: [“eom”, “leaf”]
- For all subsequent “secondary” clustering algorithms:
    - “max_cluster_size”: [0.1 * N, 1.0 * N]
    - “min_cluster_size”: [0.0 * N, 0.1 * N]
    - “min_samples”: min(128, [0.0 * N, 0.01 * N])
    - “alpha”: primary: [0.1, 5.0]
    - “cluster_selection_method”: [“eom”, ”leaf”]

Where N is the number of samples being clustered. Note that “min_samples” was capped to 128 to avoid out-of-memory errors.

Classification Hyper-Parameters Fine-Tuned with Optuna

Model hyper-parameters for CT-level label classification algorithms in Experiment 2 were optimized with Optuna, with the following setup:

- algorithm taken from ["ridge", "lasso", "elasticnet"]
- if algorithm == "ridge":
    - scikit-learn algorithm = RidgeClassifier
    - alpha taken from (0.01, 10.0)
- if algorithm == "lasso":
    - scikit-learn algorithm = LogisticRegression

    - penalty = "l1"
    - solver taken from ["liblinear", "saga"]
    - C taken from (0.01, 10.0)
- if algorithm == "elasticnet":
    - scikit-learn algorithm = LogisticRegression
    - penalty = "elasticnet"
    - solver = "saga"
    - C taken from (0.01, 10.0)
    - l1_ratio taken from (0.0, 1.0)
