# Supplementary material for: Analysis of eligibility criteria clusters based on large language models for clinical trial design
Source: J Am Med Inform Assoc. 2024 Dec 26;32(3):447–58. doi: 10.1093/jamia/ocae311 (PMC11833473; doi:10.1093/jamia/ocae311)
Supplement: ocae311_Supplementary_Data [file ocae311_supplementary_data.zip › ocae311_Supplementary_Data/Supplementary Information S3.pdf]

S3 – Prompts Used Throughout the Study

GPT-3.5 Turbo Prompt for Cluster Label Generation

The prompt used to generate human-readable labels for each cluster by prompting GPT-3.5 Turbo was the following:

```
I have a topic that contains the following documents:

[DOCUMENTS]

The topic is described by the following keywords:

[KEYWORDS]

Based on the information above, extract a short but highly descriptive topic label of at most 5 words.

Make sure it is in the following format: <topic type>: <topic label>, where <topic type> is either "Inclusion criterion: " or "Exclustion criterion: "
```

Where [DOCUMENTS] was replaced by the raw text of a representative sample of 20 eligibility criteria from a given cluster, and [KEYWORDS] was replaced by the cluster keywords identified by our adapted BERTopic pipeline. Note: the output of GPT 3.5-Turbo given this prompt was only used for visualization and interpretability, and not for evaluating cluster quality.

GPT-3.5 Turbo Prompt for Eligibility Section Generation

The prompt used in Experiment 3 to generate eligibility criterion sections by prompting GPT-3.5 Turbo with CT data was the following:

```
I have a clinical trial that includes the following information:

[CT_DATA_TEXT]

Based on the information above, generate the eligibility criteria section for this clinical trial.

Make sure the generated section includes [NUM_ELIGIBILITY_CRITERIA] eligibility criteria and has the following format:

Inclusion criteria:

<all inclusion criteria>

Exclusion criteria:
```

<all exclusion criteria>

```

Where [CT_DATA_TEXT] was replaced by the content of a CT from which only the eligibility criterion module was removed, and [NUM_ELIGIBILITY_CRITERIA] was replaced by the average number of eligibility criteria per CT in the run condition type (21 for C01, 30 for C04, 21 for C14, and 24 for C20).

## GPT-3.5 Turbo Prompt for Computing Ceiling Performance in Eligibility Section Generation

The prompt used in Experiment 3 to reformulate eligibility criterion sections by prompting GPT-3.5 Turbo to compute ceiling performance was the following:

```
You will be provided with an eligibility criterion section from a clinical trial.

Your task is to reformulate the section while preserving its meaning, intent, and key details.

The reformulated text should be written clearly and concisely, with professional and formal language suitable for clinical documentation.

Instructions:

Make sure the generated section includes [NUM_ELIGIBILITY_CRITERIA] eligibility criteria.

The list of criteria can be written in a different order than the original one.

Ensure that the reformulated section contains the same details and structure as the original but uses different wording.

Do not introduce, omit, or alter any medical or procedural information from the original text.

Write the reformulated text as a single cohesive section that maintains the technical tone and clarity expected in clinical trials.

Here is the original eligibility criterion section:

[EC_SECTION]

Provide your reformulated section below:
```

Where [EC_SECTION] was replaced by the reference eligibility section of the evaluated CT, and [NUM_ELIGIBILITY_CRITERIA] was replaced by the average number of eligibility criteria per CT in the run condition type (21 for C01, 30 for C04, 21 for C14, and 24 for C20).
```
