# Supplementary material for: Analysis of eligibility criteria clusters based on large language models for clinical trial design
Source: J Am Med Inform Assoc. 2024 Dec 26;32(3):447–58. doi: 10.1093/jamia/ocae311 (PMC11833473; doi:10.1093/jamia/ocae311)
Supplement: ocae311_Supplementary_Data [file ocae311_supplementary_data.zip › ocae311_Supplementary_Data/Supplementary Information S4.pdf]

S4 – Cluster Comparisons for Other Condition Filters

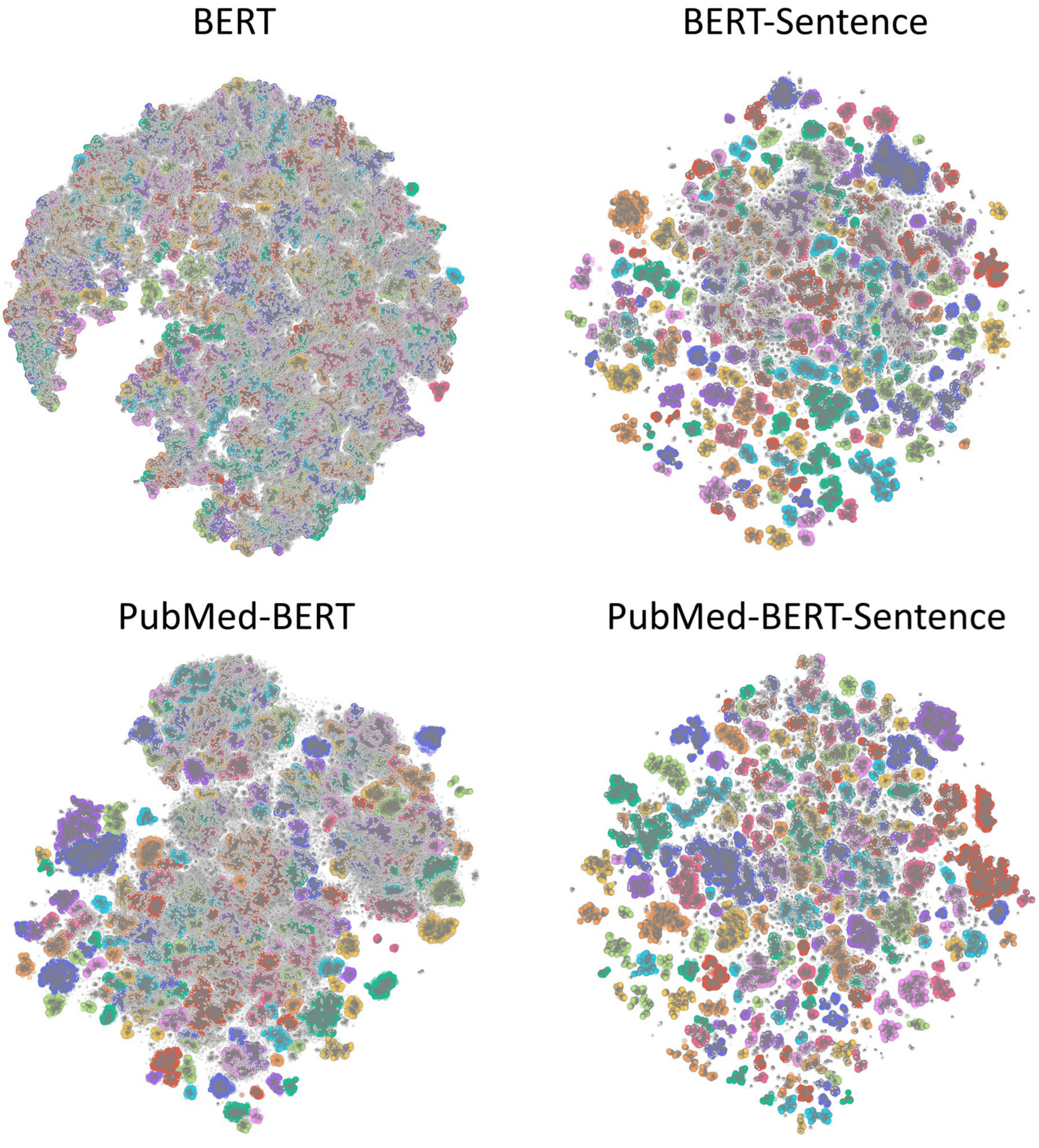

Figure A. Clusters extracted from CTs with at least one condition starting with C04 (Neoplasms). Colors represent different clusters, and grey dots are eligibility criteria with no defined cluster. N_{ECs}: 326,866; N_{CTs}: 14,985; $N_{clusters}$: {BERT: 358, BERT-Sentence: 247, PubMed-Bert: 242, PubMed-BERT-Sentence: 221}.

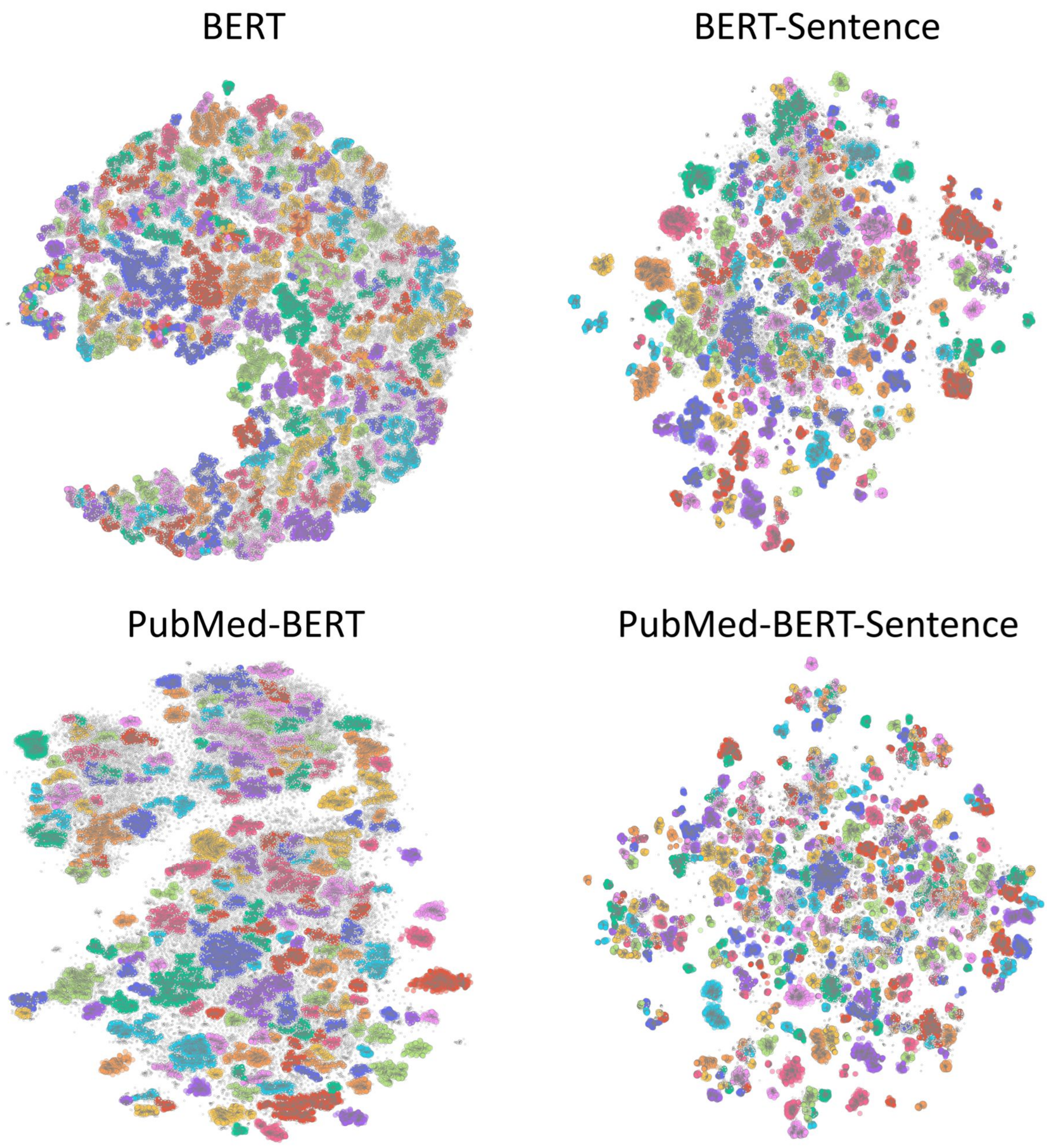

Figure B. Clusters extracted from CTs with at least one condition starting with C14 (Cardiovascular Diseases). Colors represent different clusters, and grey dots are eligibility criteria with no defined cluster. N_{ECs}: 87,948; N_{CTs}: 5,293; $N_{clusters}$: {BERT: 332, BERT-Sentence: 258, PubMed-Bert: 198, PubMed-BERT-Sentence: 454}.

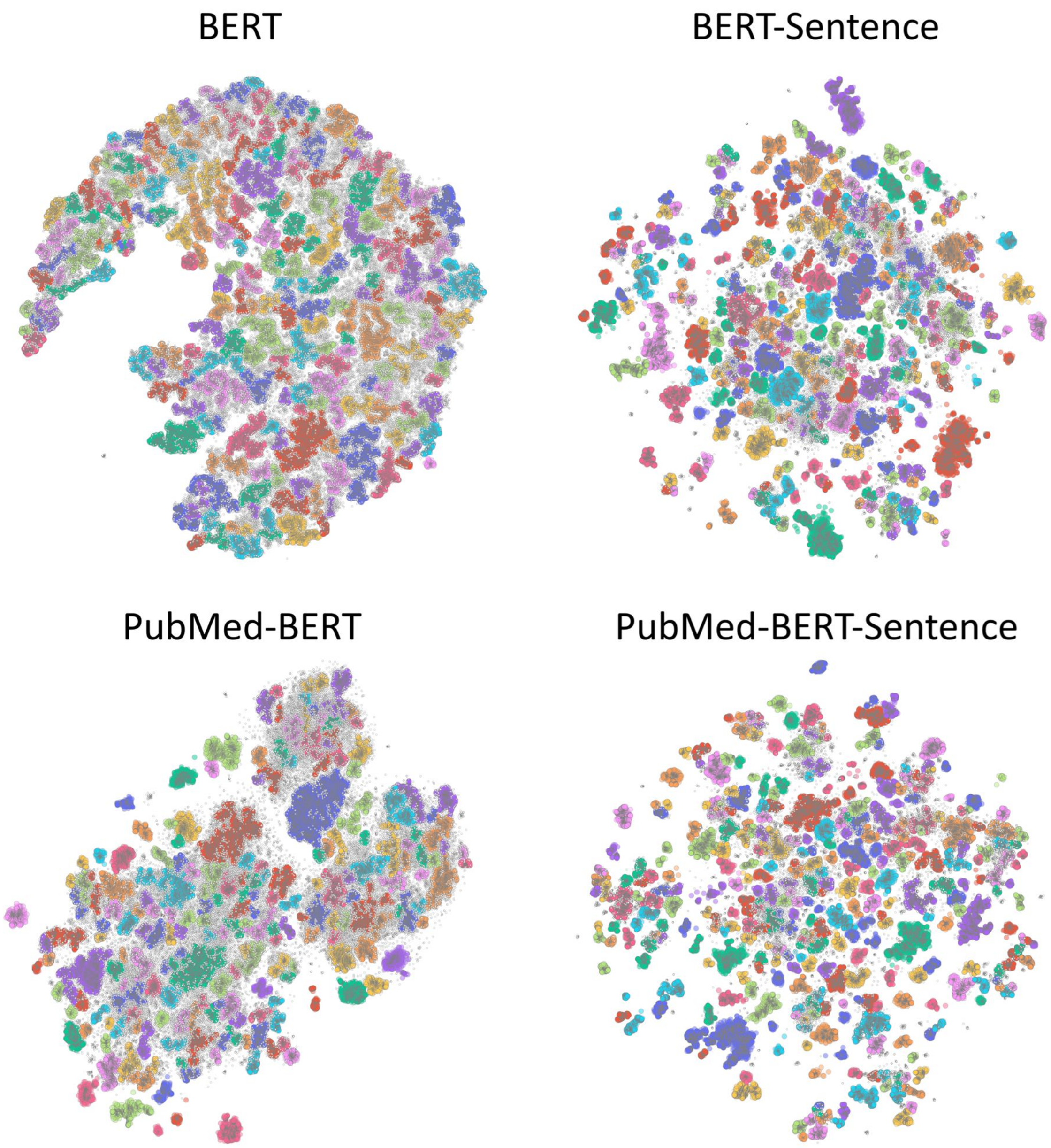

Figure C. Clusters extracted from CTs with at least one condition starting with C20 (Immune System Diseases). Colors represent different clusters, and grey dots are eligibility criteria with no defined cluster. N_{ECs}: 102,698; N_{CTs}: 4,873; $N_{clusters}$: {BERT: 252, BERT-Sentence: 302, PubMed-Bert: 232, PubMed-BERT-Sentence: 352}.

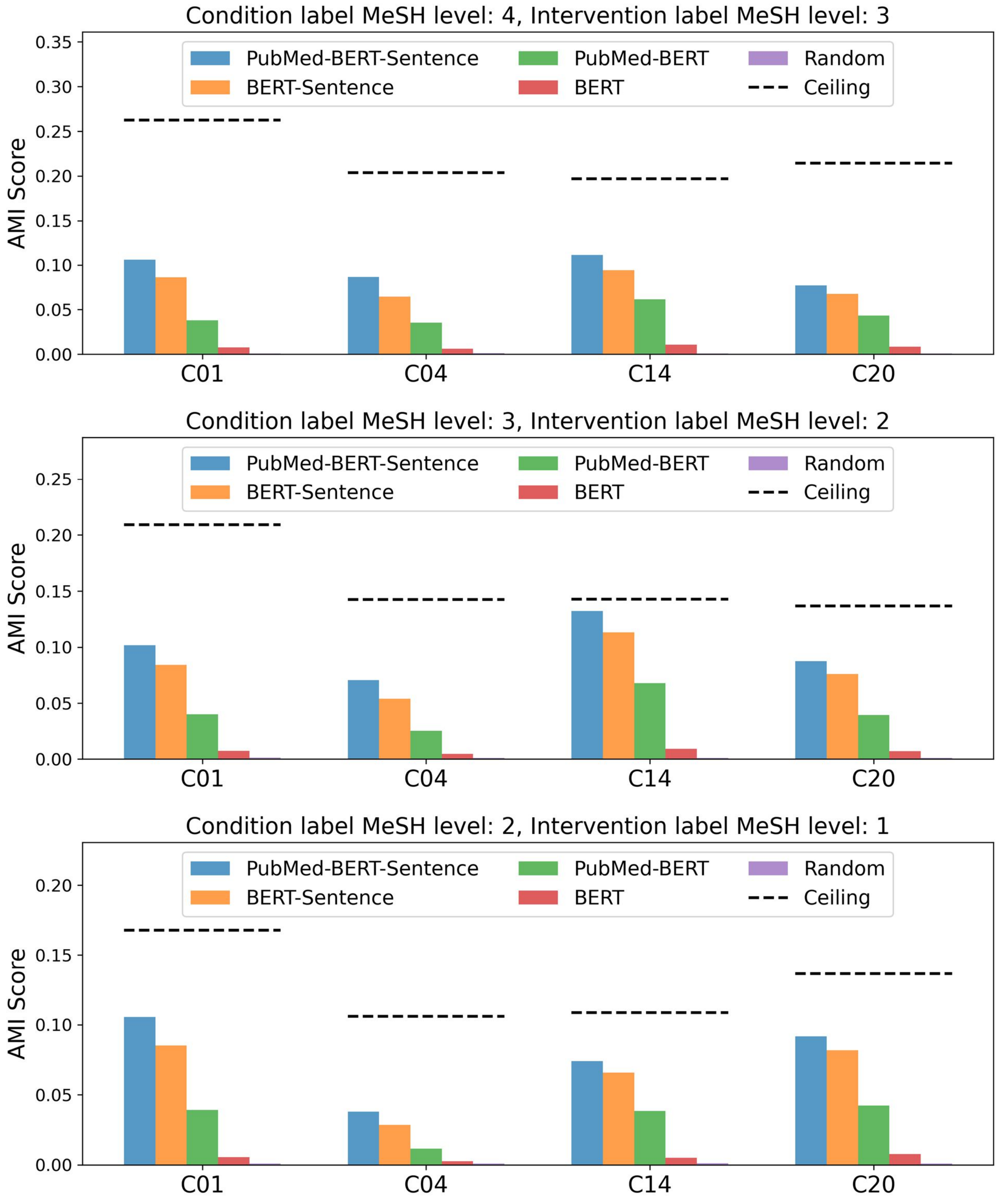

Figure D. Absolute AMI scores (i.e., not normalized with ceiling performance) for alignment with CT-protocol labels across different levels of label granularity and condition types. C01 – Infections; C04 – Neoplasms; C14 – Cardiovascular Diseases; C20 – Immune System Diseases.
