# Supplementary material for: Analysis of eligibility criteria clusters based on large language models for clinical trial design
Source: J Am Med Inform Assoc. 2024 Dec 26;32(3):447–58. doi: 10.1093/jamia/ocae311 (PMC11833473; doi:10.1093/jamia/ocae311)
Supplement: ocae311_Supplementary_Data [file ocae311_supplementary_data.zip › ocae311_Supplementary_Data/Supplementary Information S6.pdf]

S6 – Cluster Output Details

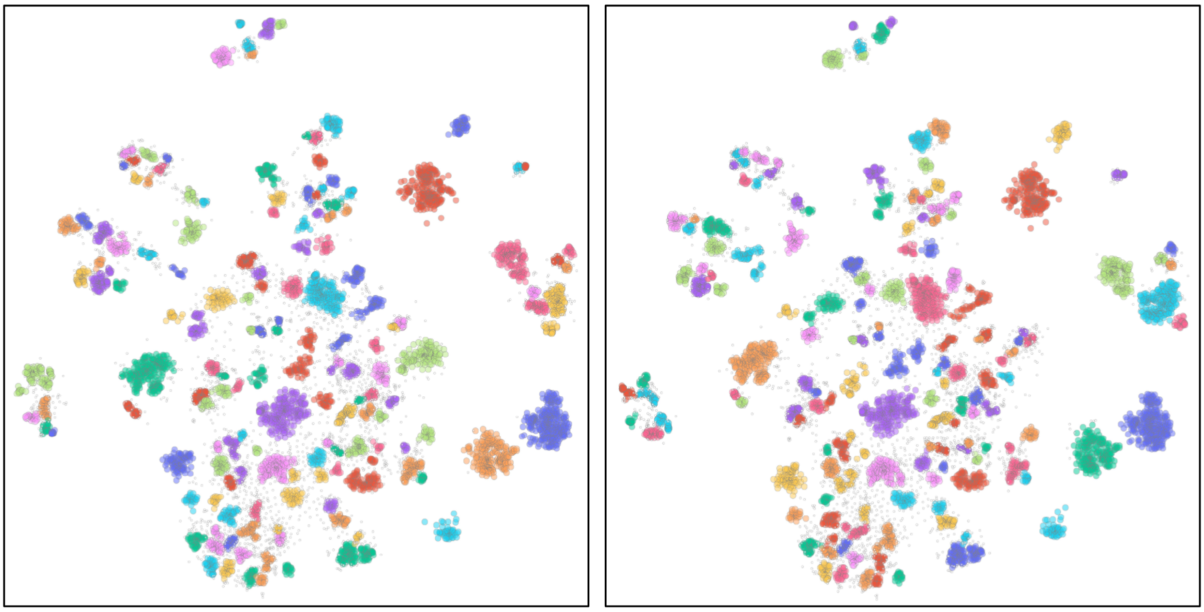

Figure J. Comparison of the output of different runs (left – run 1, right – run 2) of the clustering pipeline for eligibility criteria extracted from all CTs with similar phase, condition(s), and intervention(s) as a target CT in Phases 1/2 treating cutaneous T-cell lymphoma with oral bexarotene and photopheresis, embedded with PubMed-Bert-Sentence. Both runs give very similar outputs. Note that cluster colors have no particular meaning and should not be accounted for differences between both runs. Both runs can be visualized interactively from the HTML visualizations[1] "visualizations_exp_3/example_with_CT-04_B" and "visualizations_exp_3/example_with_CT-20_C" (conditions in the target CT include both a condition of type C04 and another of type C20).

[1] https://minhaskamal.github.io/DownGit/#/home?url=https://github.com/ds4dh/eligibility_criterion_clustering/tree/master/visualizations

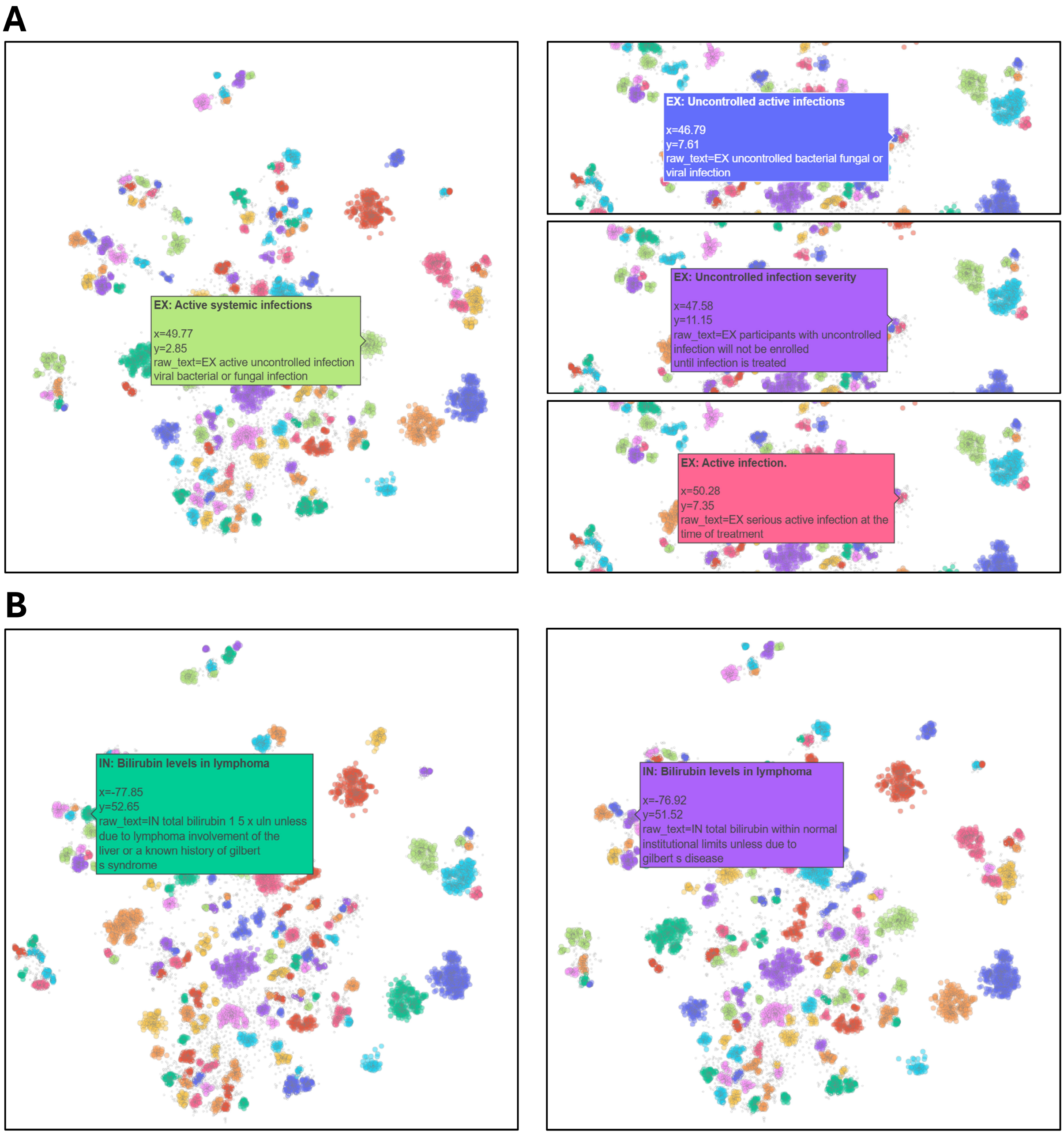

Figure K. Detailed comparison of different runs for the visualization shown in Figure J (left – run 1; right – run 2). **A.** Case where clusters are different between runs (run 2 separates the cluster of run 1 into subclusters). This is the rarest case. **B.** Case where clusters correspond. This is the most common case. Full interactive visualization are available for download (link in the caption of Figure J).

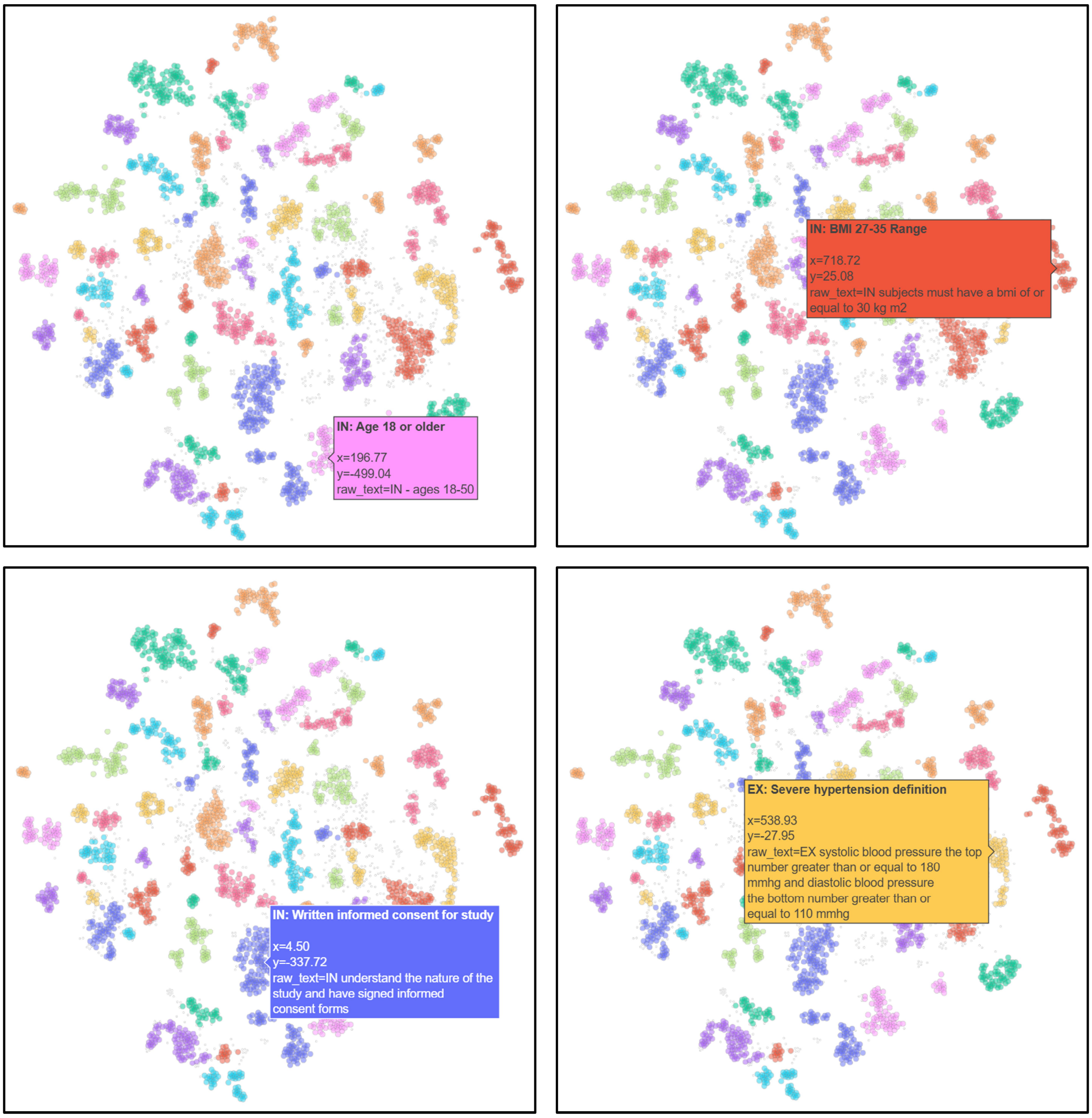

Figure L. Example of different clusters for eligibility criteria extracted from all CTs with similar phase, condition(s), and intervention(s) as a target CT in Phase 4 clinical trial that treats hypertension with valsartan or amlodipine, embedded with PubMed-BERT-Sentence. We chose to highlight eligibility criteria with rather universal topics to show they fall in different clusters, but more specific ones can be visualized and navigated interactively from the HTML file available for download (link in the caption of Figure J; see the file “visualizations_exp_3/example_with_CT-14_A”).
